# Supplementary material for: Are carbon emissions trading and green financial instruments synergistic? -Comprehensive quantitative research based on content analysis
Source: PLoS One. 2024 Mar 7;19(3):e0298601. doi: 10.1371/journal.pone.0298601 (PMC10919601; doi:10.1371/journal.pone.0298601)
Supplement: S1 Data — (ZIP) [file pone.0298601.s001.zip › Data/地级市Z_F工作报告.zip!/║╙▒▒/▓╫╓▌╩╨/└┤╫╘╣1⁄2╓┌║┼í╛┬φ┐╦╩2╛▌═°í┐_2006.docx]

现在，我代表沧州市人民政府，向大会作工作报告，请各位代表审议，并请各位政协委员和列席会议的同志提出意见。

一、“十五”时期和2005年工作回顾

过去五年，是我市改革和发展进程中不平凡的五年。在省委、省政府和市委的坚强领导下，我们紧紧团结和依靠全市人民，坚持发展第一要务，积极地、有作为地贯彻国家宏观调控政策，成功战胜非典疫情、禽流感和各种自然灾害，有效克服改革和发展中不稳定、不健康因素，经济建设和社会发展取得了令人瞩目的好成绩，圆满完成了“十五”计划确定的各项工作任务。

“十五”时期，是我市发展史上成就显著的时期。经济持续快速发展。到“十五”末，地区生产总值累计完成3504.5亿元，年均增长12.4%（同口径）。全部财政收入累计完成285.9亿元 ，年均递增16.4%；项目建设取得重要突破。“十五”期间，累计完成全社会固定资产投资1062.5亿元，年均增长14.4%。黄骅大港建成通航，朔黄铁路复线、京沪、石黄（沧石段）高速公路建成通车，TDI“2改3”、沧炼综合技术改造、华北石化10万吨聚丙烯等一批项目相继竣工投产；各项改革不断深化。成功进行了农村税费改革、财政体制改革和粮食流通体制改革，国有企业改革取得重大进展；对外开放步伐明显加快。五年来，实际利用外资4.33亿美元，其中直接利用外资2.93亿美元。华油螺旋钢管、黄骅聚鑫五金、肃宁美华纺织、瀛海香料等一批独资、合资项目相继签约和开工建设；城乡面貌变化明显。农业和农村经济进一步发展，产业化经营水平有了新的提高。先后实施了一批完善城市功能、改善城乡人民生活的给排水、燃气、供热、供电、通讯、住房等重点工程。较好地解决了人畜饮水安全和农村道路“村村通”等问题，城乡居民生活环境发生巨大变化；社会事业全面发展，人民生活水平显著提高。国民教育体系逐步完善，义务教育、职业教育、高等教育全面发展。低生育水平得到巩固，人口自然增长率控制在7‰以内。农村新型合作医疗试点稳步推进，城乡群众医疗卫生条件不断改善。造林绿化步伐明显加快，环境保护得到加强，城市大气污染、重点流域水污染防治效果明显。2005年城市居民人均可支配收入、农民人均纯收入分别比2000年增加3045元和782元，五年平均增长9.1%和5.6%。

2005年，我们紧紧围绕建设全省经济举足轻重增长极的目标，倾心打造两大战略重点，合力突破三条战线，经济社会呈现出健康快速协调发展的好势头，为我市稳步跨入发展的快车道和全面开启“十一五”新篇章提供了强劲动力。

（一）经济继续保持高位运行，综合实力明显增强。完成地区生产总值（含华北油田）1083亿元，是2000年（现价）的2.3倍，比上年增长18%。一、二、三次产业增加值分别完成133亿元、652亿元和298亿元，比上年增长4.8%、23.3%和12.9%。工业生产呈现强劲增长势头，全市规模以上工业完成增加值385.3亿元，增长34.5%，增速居全省第一 。全部财政收入完成82.9亿元，增长27.3%，其中一般预算收入31亿元，增长25.7%，经济增长质量有了新的提高。

（二）临港区域发展步伐加快，中心城市经济圈建设顺利启动。成立了临港区域经济协调发展委员会和加快中心城市发展委员会；编制了港口总体发展规划、产业发展规划、临港化工园区规划和港口城市规划；开展了5万吨、10万吨级码头前期工作和20万吨级航道建设的可行性研究和论证。总投资22亿元的综合港区前期准备工程已经开工，国华沧东电厂、外航道整治、液体化学品码头、黄骅港二期工程等重点项目顺利实施，3万吨甲乙酮、成龙玻璃等新型工业项目相继建成投产。黄骅港全年完成货物吞吐量6781万吨，跻身全国十大港口行列。着力打造以“三区一县”为主的中心城市经济圈，理顺管理体制，调整区域布局，搭建发展平台，各项工作如期推进。

（三）“三条战线”全面突破，各项工作成效显著 。项目建设进展顺利。重点项目个数明显增加，投资总量和项目质量大幅提高。全社会固定资产投资完成354.5亿元，增长45.5%，年增投资超过百亿元。全市投资1000万元以上重点项目达到1917个，其中，竣工项目466个，年内新开工项目422个，续建项目244个。津汕、保沧、石黄（沧港段）三条高速公路全面开工，黄骅港外航道整治工程顺利完成，华北石化10万吨聚丙烯项目竣工投产，沧东电厂一期、华北石化500万吨炼油及10万吨聚苯乙烯项目进展顺利。12万吨己内酰胺、5万吨TDI、10万吨DNT、华峰热电等重大项目前期工作取得重要进展。

国企改革取得重大成就。2005年是国企改革享受国家优惠政策的最后一年，市政府不惜举债5.9亿元，对95家市属国有企业实施改革攻坚。市属35家国有工业企业改革基本完成，60家商业企业改革快速推进。对市亚胺厂等5家企业实行破产接收，对化机集团、造纸厂等18家企业依法实施破产重组，对大化集团、沧化集团等12家企业，实行整体改制或资产重组，中盐长芦沧州盐业集团正式挂牌运营。

城市建设成效显著。投资28.2亿元，实施了“一拓三通”等七项城建重点工程，是历年来城市建设投资最多的一年。迎宾大道、御河路西段、黄河路西段、浮阳大道南段建成通车；南湖公园改造及水系整治工程，市民关注的热点工程，公共医疗卫生工程等均取得了积极进展。南湖确权、摄影大世界、华北商厦二期、小孙庄拆迁改造等遗留问题得到较好解决。城管体制及公用事业改革稳步推进，组建了沧州市供水排水集团公司。强化了两区城管职能，开展了城市环境综合治理活动，对市区违法建筑物私搭乱建、摊点乱摆乱设、垃圾乱扔乱倒、车辆乱停乱行、牌匾乱贴乱挂等严重影响城市环境的“五乱”顽疾，进行大规模、大力度的综合治理。到年底，已拆除违章建筑11.7万平方米，治理违章摊点1.25万个，清除垃圾2.5万立方米，城市秩序和环境卫生面貌明显改善，赢得了社会各界和市民群众的广泛赞誉。

（四）利用外资增速加快，对外开放取得积极成果。进一步加大招商引资力度，直接利用外资数额再创新高。到年底，全市直接利用外资1.44亿美元，比上年增长74%，引进省外资金55亿元，增长45%。献县泰乐铸造、安世通纺织、肃宁普华纺织、青县石油钻采仪器设备、黄骅新业石油化工、海兴滨海新城等投资超亿元的外资项目相继签约或开工建设。对外贸易持续增长，全年完成外贸进出口总额8亿美元，其中，完成出口6.1亿美元，增长26.2%。对外经济合作不断加强，签定境外承包工程和劳务合同额1000万美元，外派出国劳务1050人次。沧州海关建成开关，黄骅港被批准为一类口岸顺利开放，为进一步扩大对外贸易创造了有利条件。

（五）县域经济不断壮大，农村经济持续发展。按照突出比较优势，营造县域特色的思路，积极培育壮大县域主导产业，县域经济实力进一步增强。弯头管件、五金加工、汽车模具及铸造、汽车摩托车零配件、包装机械、线路板、裘皮纺织等产业发展迅速，特色突出，亮点凸显。中原、宏润、兴林等一批新建项目相继建成投产，产业集聚作用日益显现，成为拉动县域经济快速发展的重要力量。认真落实各项惠农政策，减轻农民负担，保护和发展种粮农民的积极性。先后从岳城水库、王快水库调水2.1亿立方米，有力地支持了农业生产。全年粮食总产量达到362.1万吨，比上年增产38.4万吨。完成植树造林41万亩，森林覆盖率提高1.5个百分点，有效改善了生态环境。认真做好禽流感防治工作，畜牧养殖业平稳发展，全年完成牧渔业产值90亿元，占农业总产值的份额提高到41%，产业结构进一步优化。代表省有关部门制定了水貂、狐狸屠宰加工等7个省级地方标准，培育了“小洋人”、“宏达”等一批中国驰名商标和省内知名企业，龙头企业带动作用明显增强，农业产业化水平进一步提高。

（六）就业和社会保障工作稳步推进，人民生活水平明显提高。实施积极的就业政策，进一步完善失业保险与再就业联动机制，“两个确保”和“社会低保”工作得到切实加强。归集住房公积金16亿元，向1000多户城镇最低收入家庭提供了住房租金补贴。5.8万名城镇贫困居民全部落实城镇低保，农村低保全面启动。加大扶贫工作力度，争取省以上扶贫资金1.56亿元，贫困村和贫困人口自我发展能力得到较快提高。新建、改建和维修农村危桥158座，完成了188个村、361.7公里的农村道路“村村通”工程，解决了158个村、16万人口的饮水安全问题。2005年，城市居民人均可支配收入8593元，增长16.4%；农民人均纯收入3300元，增长9.2%。全社会消费品零售总额完成250.1亿元，比上年增长15.1%，住房、汽车、通讯、旅游成为新的消费热点。

（七）社会事业健康发展，和谐社会建设取得积极进展。科技体制改革进一步深化，科技创新能力持续提高。对10个重点产业技术难题开展了技术攻关，高新技术企业发展到122家。新建扩建农村中小学110所，对农村中小学贫困生实行“两免一补”，受资助人数达14.9万人。市中心医院外科大楼建成投入使用，市中西医结合医院被评为全国重点中西医结合医院。在全省率先完成市、县两级疾控中心建设，农村新型合作医疗试点取得积极进展，城乡群众医疗卫生条件不断改善。706实验台建成开播，新发展农村有线电视用户6.5万户，广播电视事业迈出新的步伐。大力弘扬特色优秀文化，杂技《女子造型》在第十届中国吴桥国际杂技艺术节上获得金狮奖。基层文化建设得到加强，群众文化活动日趋活跃。竞技体育水平明显提高，全民健身活动深入开展。水资源、国土资源、海洋资源保护取得显著效果，封存、关闭市区自备井113眼，减少开采量300万立方米/年，有效遏制了超采地下水的势头。思想道德建设不断加强，群众性精神文明创建活动深入开展，有6个单位（村、镇）获得全国文明单位（村、镇）称号。加强对危险化学品、烟花爆竹、建筑施工等高危行业的安全监管，安全生产形势总体稳定。以侦破命案和打击“两抢一盗”犯罪为重点，加大对刑事犯罪打击力度，命案破案率达95.6%，创历史新高。成功告破了市区“6·12”持枪抢劫杀人案、献县“10·17”持枪抢劫案等在全国影响较大的案件，受到省公安厅、省政法委和公安部的嘉奖。依法管理民族宗教事务，认真对待人民群众来信来访，及时化解人民内部矛盾，为全市经济社会发展创造了稳定和谐的社会环境。成功举行了“平安—05”城市救援演习，应对和处置突发事件的能力得到提高。较好地完成了征兵和优抚安置工作，“双拥”共建、国防教育和国防后备力量建设不断加强，驻沧部队在建设沧州、发展沧州中发挥了积极作用。统计、审计、物价、外事、档案、气象、残疾人等各项事业也都取得了新的成绩。

（八）民主法制建设进一步巩固，政府自身建设得到切实加强。自觉接受市人大常委会工作监督、市政协民主监督，主动听取人大代表、政协委员、各民主党派和工商联提出的意见和建议。认真办理人大代表建议和政协提案，一批人民群众关心、关注的热点、难点问题得到较好解决。完善了重大问题集体决策制度、专家咨询制度、社会公示制度和社会听证制度，依法决策、民主决策水平有了新的提高。邀请人大代表、政协委员列席市政府常务会议，充分听取人大代表和政协委员对涉及全市发展重大决策的意见和建议。在市区环境综合治理活动中，市人大、市政协支持市委、市政府决策部署，形成强大工作合力，有力地推动了市区环境综合治理活动的深入开展。积极开展提速、提质、为人民、促发展的“双提”工程，全面实施承诺践诺、首办负责、投诉受理、责任追究等工作制度，纠风工作得到国家和省纠风办的充分肯定，效能建设取得突破性进展，政务环境进一步优化，服务水平有了新的提高。认真落实机构编制管理规定，有效控制机构编制和人员增长，共精简财政供养人员16190名，完成了财政供养人员总量控制和事业单位清理整顿年度目标。

各位代表，过去五年，特别是去年，我市经济建设和社会发展取得的好成绩，是我们在历届政府工作的基础上，在省委、省政府和市委的坚强领导下，在市人大、市政协的监督支持下，全市广大干部和人民群众共同奋斗的结果。在此，我代表市人民政府，向辛勤工作在各条战线上的全市各族人民，向给予我们支持与监督的人大代表、政协委员、各民主党派、工商联、无党派人士，向大力支持我们工作的离退休老干部、驻沧单位、人民解放军和武警部队，向所有关心、支持沧州发展的同志们、朋友们，表示崇高的敬意和衷心的感谢！

各位代表，成绩令人欣慰，任务依然艰巨。面对全市人民加快发展的迫切要求，我们深感责任重大，前进道路并不平坦，发展进程中还有许多亟待克服的困难和问题，主要是：经济总量偏小，结构性矛盾尚未消除，特别是工业经济的总量和质量有待提高，振奋人心的大项目少；对外开放和环境建设仍需进一步加强；城市建设任务繁重，管理水平有待提高；农村发展、农民增收任务艰巨；就业压力较大，社会事业欠帐较多，和谐社会建设有待进一步探索；政府工作需要进一步改进和加强，政府部门工作效率有待提高。对这些矛盾和问题，我们将在发展的实践中大胆探索，逐一破解，全力推进我市经济社会的健康快速发展。

二、“十一五”时期经济社会发展的主要任务

告别“十五”，跨入“十一五”，我们已经站在了一个新的起点上。未来五年，是全面建设小康社会的关键时期，也是我市经济社会发展不可多得的时期，更是我们必须紧紧抓住的重大机遇期。

从国内外发展环境来看，“十一五”时期是机遇与挑战并存，总体发展环境向好，但不利因素可能增多的时期。经济全球化、区域经济发展，特别是京津冀都市圈、环渤海经济圈的崛起，以及天津滨海新区的开发，为我们有效发挥区位、产业、港口、交通、资源等优势提供了难得的历史机遇；在南方发达地区不同程度出现地荒、电荒、劳动力缺乏的大背景下，我们可以凭借丰富的土地资源、劳动力资源、沿海电力基地建设和港口功能的完善等资源优势，吸引更多国内外资本和产业转移项目落户沧州，为我们加快发展创造了独特的优势条件；省委、省政府对沧州的高度关注，全市干部群众高涨的发展热情，为我们加快发展提供了强有力的政策保障和动力支持。但是，我们也面临着严峻挑战。第一，在国内外经济竞争日益加剧的情况下，我们的经济结构、总量、质量还处于弱势地位。第二，尽管我们已经驶入了发展的快车道，但加快发展的基础还不稳固，仍然处在爬坡过坎的阶段。第三，经济社会快速发展，新情况、新问题不断涌现，各种利益关系交织、碰撞，使得一些深层次矛盾也进入了凸显期。能不能抓住机遇，发挥优势，扬长避短，化不利因素为有利条件，不失时机地把我市经济和社会发展推向一个更高层次，是全市人民对我们的殷切希望，也是对我们领导和推动发展能力的一次最直接的检验。因此，这一时期，我们既要向前看，抓住机遇，加快发展。又要回头看，正确处理好历史遗留问题；既要一心一意抓发展，快速扩张经济总量，提高运行质量，又要努力解决好与人民群众息息相关的问题，让全市人民更加直接地享受到改革和发展的成果。

“十一五”时期我市经济和社会发展的主要目标是：经济发展再上新台阶。地区生产总值年均增长13%以上，2010年生产总值达到2000亿元左右，人均生产总值达到2.8万元以上，超过全省平均水平；资源开发与生态环境相协调。单位生产总值能源消耗降低14.5%。农业节水灌溉面积增加50万亩，工业万元生产总值耗水量降低19%。工业固体废物综合利用率达到96%；人民生活水平得到新提高。人口自然增长率控制在8‰以内，城市居民人均可支配收入年均增长10%，农村居民人均纯收入年均增长6%以上，社会消费品零售总额年均增长12.5%以上；社会事业取得新进步。初中适龄人口入学率达到99%，高中阶段教育和高等教育毛入学率分别达到85%和25%。民主法制意识显著增强，公民思想道德素质、科学文化素质和身体健康素质有较大提高。

实现“十一五”发展目标，必须始终坚持做到以下六点：

第一，必须深入贯彻科学发展观。科学发展观是新时期发展观和方法论的集中体现，其核心是“科学发展”。要切实转变经济增长方式，积极发展循环经济、绿色经济、集约经济，实现经济增长速度与结构、质量和效益相统一。要坚持“五个统筹”，突出以人为本，在发展经济的同时，更加注重社会事业发展，更加注重区域统筹和城乡一体化，更加注重人与社会、自然和生态的和谐，使经济发展与人口、资源、环境相协调，实现经济社会的全面、协调和可持续发展。

第二，必须突出重点，快速提升经济实力。当前，我市发展的主要矛盾是解决发展不足的问题，特别是要着重解决经济总量小和运行质量低的问题。要倾力打造临港区域、中心城市、县域经济三大经济板块，以临港区域为龙头，保持对全市经济的持续拉动力，增强发展后劲；以中心城市为支撑，城乡统筹、区域统筹，增强对全市经济的辐射带动力；以县域经济为依托，强工促农、兴县富民，增强县域经济竞争能力。

第三，必须把投资拉动作为加快发展的首要选择。投资不仅是拉动经济发展的主要力量，而且也是经济结构调整的决定性因素。加快发展，调整结构，必须坚持在发展中调整，把小的做大，把大的做强，不断积聚发展能量。要紧紧抓住未来五年的时间，坚持以“项目为纲”，有质量的项目越大、越多越好，有效益的投资越多、越快越好，努力使投资保持一个较高的增长水平，使项目建设保持一个较快的发展势头，做大增量，盘活存量，扩张总量，为建设全省经济发展举足轻重的增长极奠定基础。

第四，必须把工业强市作为经济发展的主引擎。没有发达的工业作支撑，就没有城市和农村的现代化。要进一步强化工业主体地位，迅速提高工业经济对GDP、城乡居民收入、财政收入、社会就业、农村人口转移的贡献率，真正让工业经济成为强市之基，立市之本。坚持扩张总量与提高质量两手抓，围绕化工、电力能源、装备制造、纺织服装、食品加工等主导产业和优势行业，增投入，上项目，搞技改。坚持走新型工业化道路，大力发展循环经济，提高资源利用效益。引导生产要素向园区、城镇和主导产业集中，形成有利于经济集约增长的生产力布局。

第五，必须把改革开放作为加快发展的动力保障。坚持以改革的办法解决前进中的矛盾，以开放的举措推进大开发、大发展，以发展的成果改善人民群众生活。要继续深化各项改革，大力推进体制、机制创新。强化政府社会管理和公共服务职能，完善自然灾害、事故灾害、公共突发事件应急预警机制。深化国有企业和国有资产管理体制改革，活化机制，增加效益。继续把扩大对外开放作为优化经济结构、扩张经济规模、增强经济实力的主要动力和重要实现途径，以直接利用外资为重点，创新招商方式，扩大对外开放，搞活外贸经营，着力构建外向型经济格局。

第六，必须坚持统筹协调发展，推进和谐社会建设。我们发展的最终目的，就是要让全市人民更广泛、更直接、更细微地享受到改革和发展的成果。要从人民群众最直接、最关心、最现实的问题入手，加快发展社会各项事业，推进和谐社会建设。要按照城乡统筹发展的要求，加快社会主义新农村建设步伐，以产业化提升农业，以工业化富裕农民，以城市化带动农村，缩小城乡差距，改善城乡居民特别是农村人口的生产生活条件。大力发展商贸流通、物流配送、旅游观光、房地产等第三产业，满足人民群众多样性消费需求。大力推进技术创新，普及先进适用技术，加快科技成果向现实生产力转化。全面推进素质教育，完善公共卫生和社会保障体系，加大扶贫开发力度，改善贫困人口生产生活条件。

三、努力开创2006年工作新局面

2006年是“十一五”的开局之年，开创性地做好今年的政府工作，对“十一五”规划的顺利实施至关重要。

政府工作的指导思想是：以邓小平理论和“三个代表”重要思想为指导，全面落实党的十六大、十六届五中全会和省委六届八次会议精神，紧紧围绕建设全省经济发展举足轻重的增长极这一目标，以科学发展观为统领，以大发展、快发展为主基调，坚持工业强市，加大投入、加强项目建设、加快改革开放和城市化进程，着力打造临港区域、中心城市和县域经济三大经济板块，壮大经济实力，提高运行质量；坚持以人为本，统筹经济和社会协调发展，同步推进物质文明、政治文明和精神文明建设，让改革和发展的成果惠及全市人民，构建和谐沧州。

主要预期目标：地区生产总值增长14%，全社会固定资产投资增长26%，全部财政收入增长15%，城市居民人均可支配收入和农民人均纯收入分别增长11%和7%。

做好2006年各项工作，必须牢牢把握以下八个重点：

（一）合力打造三大经济板块。临港区域、中心城市、县域经济是“十一五”时期的战略重点，也是2006年的工作着力点。第一，培育临港产业带。深入谋划，精心组织，举全市之力，推进综合性大港建设。加强对外宣传，加强与冀晋陕蒙腹地有关单位和机构的联系，努力扩大港口的知名度、影响力和辐射力，增强港口对全市经济的持续拉动力。以临港化工园区、港城开发区、神华万亩工业园为中心，以沿海临港区域为纵深，推进化工产销基地、电力能源基地、加工和装备制造业基地、大型物流基地等四大产业基地建设，加快形成临港产业隆起带。第二，做强中心城市经济。高度重视并着力解决中心城区产业空心化问题，按照优先发展工业的要求，调动一切积极因素，增投入、上项目、兴产业、聚人气。“三区一县”要按照布局分工，以市经济技术开发区、市区西部高新区、东部和东北部工业区为平台，大力发展符合中心城市特点和要求的劳动密集、产业关联度强、技术含量高、消耗低、无污染的产业，把中心城市经济实力搞大做强，提高对临港区域和县域经济的辐射带动能力。今年，要把工业项目投入作为单列指标，对“三区一县”政府和涉及项目审批的市政府部门进行考核，力促中心城市经济实力快速提升。第三，增强县域经济综合实力。以特色行业、特色产品、规模企业为重点，扶持发展一批具有较强竞争力的优势企业和名牌产品；大力支持成长性好、竞争力强、销售收入超过一定规模的企业加快发展；鼓励企业加大投入，积极开发核心技术，向产业价值链的高端迈进。对特色行业中的优势企业，实行重点帮扶，鼓励企业研发新技术，开发新产品，提高核心竞争力。在占国内市场份额较大的产业中，力促一批企业成为全国行业的“贯标”企业；以优势企业为核心，引导中小企业向主导产业和骨干企业靠拢，形成一批“专、精、新”中小企业群体，进而带动和促进县域经济快速发展。全面落实以放宽准入、公平待遇为重点的各项政策措施，重点是放宽民间资本投资领域和经营条件，消除各种歧视性规定，破除各种非政策壁垒。健全服务民营企业发展的运行机制，提高民营经济在县域经济中的份额，强化民营经济的支撑作用。

（二）强力推进项目建设。按照项目建设与区域发展相协调，与资源条件相适应，与环境容量相匹配，与产业整合相结合的原则，积极谋划和建设一批关联度高、带动能力强的大项目。第一，切实抓好项目谋划。围绕临港产业、化工产业、装备制造业、高新技术产业，谋划一批有利于生产力布局调整的重大产业项目；围绕弯头管件、包装机械、电线电缆、机车配件、裘皮纺织、五金制品等特色行业，谋划一批产业集群发展项目；围绕农业增效、农民增收，谋划一批产销一体化的农业产业化项目；围绕电力能源、港口设施、道路桥梁、给排水等设施建设，谋划一批基础设施建设项目；围绕改善群众生活，谋划一批科技教育、医疗卫生、文化体育和广播电视等社会事业项目。第二，加快项目建设进度。进一步加大对项目建设的支持力度，为业主单位提供全方位优质服务。要特别加强对重点项目的支持，对重大项目要有专门班子跟踪服务，及时解决项目建设中遇到的各种困难和问题。实行项目建设激励机制，充分调动各级各类企业增投入、上项目的积极性。认真抓好618个投资1000万元以上续建和新开工项目建设，确保5万吨TDI、华油500万吨炼油、12万吨己内酰胺、3万吨三聚氰胺、华峰热电、市中心医院扩建等重大项目建设取得实效。第三，强化项目督导。要按照储备项目抓跑办、开工项目抓进度、建成项目抓达产的要求，加强协调调度和跟踪督办，力促建设项目早日投产达效，为拉动全市发展，打造全省经济增长极奠定基础。

（三）着力抓好工业经济运行。研究制定鼓励工业发展的政策措施，建立促进工业持续增长的动力机制，努力扩大增量，盘活存量。一是继续深化国有企业改革。全面完成市属95家国有企业改革扫尾工作，彻底实现职工身份和产权的双置换。积极推进大化集团等重点企业的战略合作与重组。进一步完善国有资产管理体系，加快建立国有资产运营公司，搞好破产企业的资产处置和金融不良债权回购工作。二是加强企业内部管理，向管理要效益。加快建立现代企业制度，加强企业家队伍建设，推进制度创新、管理创新，有机整合人力、物力和财力资源，降低资源消耗，促进成本节约，提升效益水平。三是扶优扶强，培育骨干、名牌企业。实施名牌战略和百家优势企业重点扶持工程，加快建立以企业为主体、市场为导向、产学研相结合的技术创新体系，支持企业用先进适用技术改造传统工艺，增强企业自主创新能力和市场竞争能力，使更多企业步入以技术造就品牌，以品牌占领市场，以市场引领企业发展的良性循环轨道。四是加强服务，搞好生产要素调节。政府职能部门要勇于开拓，善于创新，用足用好上级政策，探索服务企业发展的新措施、新办法，严肃查处干扰企业生产经营活动的乱检查、乱收费、乱罚款行为，为企业发展营造良好的外部环境。工业经济部门和经济调控部门，要搞好全市主要企业要素资源需求分析，主动加强与有关方面沟通协调，抓好煤、电、油、气、运等生产要素的运行调节，最大程度地满足企业生产需要。

（四）努力扩大对外开放。在对外开放取得长足进步的基础上，进一步提高招商引资的针对性和实效性。一是着力提高招商引资水平。以直接利用外资为重点，全面推行以商招商、代理招商、产业链招商、一对一招商、产权转让、企业并购等新型招商方式，深入开展面向国内外的招商引资活动，吸引更多国内外资金、项目落户沧州。二是锁定重点，开展好三个层次的招商活动。充分发挥比较优势，面向世界500强、国内500强和日韩台大企业、大公司、大财团，引进资金、技术和产业转移项目；抓住国内外产业转移、资本流动、要素重组的有利时机，利用港口、能源、土地综合优势，面向长三角、珠三角、山东半岛，展开全领域招商；搞好与京津基础设施、产业和技术对接，参与区域经济分工协作，承接京津辐射。要特别注意引进和建设与天津滨海新区开发相适应的产业互补和产业接替项目，以产业互补和融入产业链等形式，积极参与天津滨海新区开发。三是搭建招商平台。加强开发区、工业园区软硬环境建设，抓好各开发区的整体包装和对外推介，建立日韩台工业园，推进土地成片开发，逐步形成产业聚集效应。积极推动企业上市，拓展资金来源。四是实施“四个一百”工程。谋划100个有针对性的招商大项目；选择100家优势企业与国内外大企业、大集团进行对接；培养100人的专业招商队伍；开展百日招商活动，确保大项目招商不断档。五是搞活内外贸易，增强对经济发展的拉动能力。全面放开进出口经营权，支持出口龙头企业扩规增效。巩固扩大裘革、抽纱、餐具等传统出口基地，培育扶持化工、线缆、弯头管件等新的优势出口产业。整合资源，优化布局，完善市区三级商业网点体系，构建开放型、互联互通型物流配送网络。大力发展连锁经营、物流配送、电子商务等新型业态，积极推进生产资料市场、专业批发市场和农副产品市场建设，充分发挥商贸流通业拉动内需的作用。

（五）切实加强城市建设和管理。围绕完善功能、提高品位、展示形象，进一步加强城市建设和管理，改善人居环境，提升城市综合竞争力。一是集中力量搞好重点工程建设。着力抓好总投资30亿元的七项城建重点工程，即：“一纵三横”道路工程（经四大街、解放西路新区段、解放东路开发区段和黄河东路）；迎宾大道及关闭自备井配套工程；报业大厦、绿洲大厦等重点建筑物工程；水月寺大街改造等热点工程；一线及七条道路绿化工程；城中村改造工程；鼓楼小区、盛泽花园等精品生活小区工程。二是加强城市管理，优化城市环境。改善市容环境卫生面貌，是广大市民的共同心声，也是提高市民文明素质和生活质量，提升城市竞争力的必然要求。继续深入开展市区环境综合治理活动，充分发挥新华、运河两区政府，以及建设、土地、规划、公安、工商等部门的职能作用，开展城市管理综合执法，巩固和扩大治理成果。搞好城市出入口和重要节点的设计包装，擦亮城市名片，打造良好的城市形象。对治理后的主次干道进行硬化、绿化、美化，做到拆一条净一条，治一条变一条，为全市人民打造一个方便舒适、秩序井然、清新靓丽的新沧州。三是推进城市管理改革，搞好城市经营。树立经营城市理念和投入产出意识，深化城市投融资体制改革，全面放开搞活城市建设市场。认真抓好绿化、公交、市政、环卫、燃气、供排水等公用事业改革，降低运营成本，提高质量效益。严格垄断土地一级市场，完善土地供给和地价形成机制，盘活存量资产，激活房地产市场。

（六）认真做好“三农”工作 。按照农村工业化、农业产业化和农民城镇化的思路，高度重视并逐步解决“三农”问题，扎实推进社会主义新农村建设。一是加强农业项目建设。抓住中央财政向农村倾斜的有利时机，深入谋划一批农业产业化项目和农田水利、道路桥梁等基础建设项目，搞好项目策划包装，争取上级政策和资金支持，以农业项目建设带动农村全面发展。二是大力发展现代农业。以发展绿色农业为目标，加快农业科技进步，推行标准化生产，优化农业结构，提高农业综合效益。根据市场需求，在稳定粮棉生产的基础上，大力发展林果、蔬菜和畜牧水产养殖业，提高农副产品市场占有份额和市场竞争能力。大力推进农业产业化，发展壮大产业化龙头企业，提高产业化经营水平。健全农业技术推广、农产品市场、农产品质量安全和动植物病虫害防控体系，做好重大动物疫病防控工作，发展高产、优质、高效、生态、安全农业。三是深化农村综合配套改革，加快发展农村公共事业。落实好对农民的补贴政策，完善农民负担监督管理机制，增加农民收入。稳步推进县乡机构改革、农村义务教育和县乡财政体制改革，增强农村发展动力。依法保护耕地，完善土地征用办法，探索建立失地农民基本生活保障和就业制度。发展农村文化教育事业，普及和巩固农村九年义务制教育。强化基层文化馆、图书馆建设，引导和推动农村文化活动健康开展。加强文明生态村建设，建立健全农村公共卫生和基本医疗服务体系，实施食品药品放心工程、“万村千乡”市场工程，改善农村人口生活质量。四是有序组织农村劳动力转移和对外劳务输出。积极改善农民就业环境，解决好进城务工人员公共卫生、文化生活、子女就学、社会保障等方面的实际问题。加强农民就业转移技能培训和就业指导，提高劳务输出组织化程度，创建劳务品牌。五是搞好扶贫开发，组织实施整村推进扶贫规划，推动产业化扶贫，改善贫困村和贫困人口生产生活条件。

（七）统筹发展社会各项事业。大力发展科技、教育、文化、医疗卫生、就业、社会保障等社会各项事业，不断满足人民群众日益增长的物质文化需求。一是优先发展科教文化和体育事业。加强人才培养，完善人才引进、使用、管理的激励机制。强化科技服务、技术创新和对外交流功能，着重抓好生产力促进中心、星火科技专家大院和产学研基地建设。全面落实以县为主的教育管理体制，积极推进义务教育均衡发展。以提高教育质量为核心，扩大教育开放，大力发展普通高中教育和学前教育。结合我市产业发展需要，重点发展职业教育，推进重点职业学校建设，争取职技学院进入省级示范性学校行列。沧州师专泊头分校完成升专评估任务，沧州师专升本工作年内取得突破性进展。实施精品文化工程，打造沧州特色文化品牌。深入谋划市广电中心建设项目，加快农村有线电视“村村通”进程。切实抓好西体育场改造、沧州体育馆等项目建设，确保全民健身中心投入使用。二是加强医疗卫生和计划生育工作。深化卫生体制改革，完善公共卫生和医疗服务体系，大力发展城市社区和乡村卫生服务组织，增加肃宁、河间两个农村新型合作医疗制度试点。启动市中心医院内科大楼、市医院综合住院楼和市中西医结合医院红十字病房大楼建设，改造2所县级医院、36所乡镇卫生院，提高装备水平和医疗服务水平。加快建立计划生育利益导向机制，全面落实农村部分计划生育家庭奖励扶助制度，平抑第四次人口生育高峰。三是实施积极的就业政策，建立经济增长与扩大就业的协调推进机制，努力改善就业和创业环境，提高城乡人口就业率，确保年内新增就业3万人。高度重视大中专毕业生就业工作，确保就业率达到80%以上。四是深化行政管理体制改革。加强机构编制管理，加大事业单位人事制度改革力度，搞好事业单位清理整顿，全面完成财政供养人员总量控制目标，建立机构编制和财政供养人员总量控制长效管理机制。稳步推进水管体制、城管体制和文化管理体制改革，提高管理水平和工作效能。

（八）积极推进和谐沧州建设。按照民主法治、公平正义、诚信友爱、充满活力、安定有序、人与自然和谐相处的要求，积极推进和谐沧州建设。一是以非公有制企业、灵活就业人员、农民工、困难和破产改制企业职工为重点，进一步扩大社会保险覆盖范围，完善社会保障体系。健全城镇居民最低生活保障制度，全面启动农村低保制度，完善五保供养制度，积极推进农村养老保险制度建设。加大对困难人群和弱势群体的帮扶，建立扶助困难群众的长效机制。二是巩固和发展基层政权。认真做好第七届村民委员会换届选举工作，完善以村民委员会、城市社区居委会为主的群众自治组织，巩固基层政权，扩大基层民主，推行民主管理。要强化企业的社会责任，健全企业工会组织，完善职工代表大会制度，保障劳动者合法权益。三是大力推进政务公开，让人民群众充分享有知情权、参与权和监督权。进一步做好民族宗教工作，发展民族经济，促进民族团结。高度重视信访工作，努力做好人民内部矛盾排查调处，维护社会正常生产生活秩序。四是加强社会主义道德体系建设。深入开展理想信念教育和思想政治工作，大力弘扬以爱国主义为核心的民族精神和以改革创新为核心的时代精神，加强社会公德、职业道德和家庭美德教育，营造健康向上的社会风气，巩固社会和谐基础。五是切实维护好城乡环境和社会秩序。认真抓好安全生产，特别要加强对化工企业、烟花爆竹、气站、油站等重点部位的安全监管，保障人民生命财产安全。严厉打击入室盗窃、盗抢机动车、抢夺等各种刑事犯罪，加大对火灾、中毒、群体性事件等隐患的排查力度，加强社会治安综合治理，增强人民群众的安全感。切实加大环境保护力度，继续实施好环境保护“五个一”工程，争创河北省环境保护模范城市。

四、创新政府工作，提高执政能力

经济社会的快速发展，和谐社会的构建，对政府工作提出了更高的要求。为提高政府工作效能，适应形势发展要求，市政府将2006年定为“政府工作管理年”，努力把市人民政府建设成为阳光政府、法治政府、高效政府、为民政府、廉洁政府，敢于负责的政府和人民满意的政府。

（一）牢牢抓住发展不动摇。发展是沧州人民的最高利益，沧州需要发展，沧州人民迫切要求我们加快发展。在新的一年，市人民政府全体组成人员，必须时刻反思我们的思维方式、行政方式、工作方式，经常检查制定的政策，采取的措施，开展的工作是不是真正做到了把发展作为第一要务，能不能充分调动方方面面的积极性，符不符合大多数人的愿望，把全力推进我市的大发展、快发展作为根本职责。切实强化政府责任、部门责任、领导者责任，围绕增长目标、工作任务和具体项目落实责任，建立健全责任考评机制和责任追究机制，制定问责制实施方案。把抓落实、敢负责作为政府系统干部政治品质的集中体现，把求实效、创业绩、能干事、会干事、干成事作为政府系统干部能力和才干的鉴别标准。

（二）牢记为人民服务的宗旨。人民群众是我们的衣食父母，我们的权利是人民给的，政府就是要为人民服务。新的一年，市人民政府全体组成人员必须牢记为人民服务的宗旨，把人民群众的衣食住行、安居乐业放在心上。在政务决策中深入了解群众意愿，倾听群众呼声，有效防止决策出现大的失误。在加快发展中，千方百计提高人民群众的生活水平和生活质量，努力使市人民政府的工作顺民心、合民意，真正体现以人为本。向人民群众承诺了的目标就要全力实现，答应了人民群众的事情就要坚决兑现。

（三）提高依法行政水平。新的一年，市人民政府的全体组成人员，必须严格执行市人大及其常委会的各项决议，主动接受人大及其常委会的监督，认真办理人大代表的议案和建议，悉心听取批评、意见。加强对广大公务员尤其是各级领导干部的法制教育，强化法律意识，切实依法行政。理顺执法体制，规范行政执法主体，探索试行综合执法体系，做到文明公正执法，提高行政机关的执法水平。强化执法监督，做好行政复议和行政诉讼应诉工作。坚持求真务实，优化统计环境，坚决不搞虚报浮夸，坚决不搞脱离群众的“形象工程”。同时，清理、规范、完善、健全各项制度，真正做到依法、依规管理。

（四）增强民主观念，实行透明行政。新的一年，市人民政府必须加强与市政协的联系和协商，主动接受民主监督，对涉及面广、群众关注的重大事项，决策之前充分征求各民主党派、工商联、无党派人士和社会各界的意见。建立健全规划、价格等听证会制度，积极推进政务阳光工程，主动接受新闻媒体的舆论监督，促进市人民政府决策的科学化和民主化。

（五）加强效能建设，提高行政效率。新的一年，市人民政府的组成人员，必须以“提速、提质、为人民、促发展”为座右铭，切实提高政府的工作效率和工作质量。加快电子政务建设，探索无纸化办公，切实解决会议多、文件多、应酬多和调查研究少等突出问题。要最大限度地减少审批项目，简化办事程序，对所有投资创业项目实行代办制，限期办结。坚决克服办事拖拉、推诿扯皮等不良作风，在全体公务员中倡导“人人争先、事事一流”，真正把沧州变成资本的洼地、创业的乐土。要强化政府“一盘棋”意识，保证政令畅通。

（六）切实加强廉政建设。新的一年，市人民政府的全体组成人员，必须严格执行廉洁从政的各项规定，做到任何时候都不以权谋私，切实管好自己的配偶、子女和身边工作人员。对政府采购、土地出让、工程招标、产权交易、大额资金使用等敏感问题全部公开，主动接受各方的监督检查，对个别公务员存在的“吃拿卡要，不给好处不办事，给了好处乱办事”等问题，一经查实，严惩不贷。

各位代表，全市人民盼望沧州大发展、快发展，我们大发展、快发展的各项条件已经具备。只要我们在中共沧州市委的坚强领导下，在市人大、市政协的全力支持下，全市人民上下一心，同心同德，我们就一定能够克服前进道路上的一切困难，在“十一五”的开局之年，谱写出更加壮丽的新篇章，让全市人民在沧州的经济社会发展中得到更多的实惠。
